# Enhanced proliferation tracer reveals Dorsal-Ventral asymmetry in tracheal epithelial Renewal​

**DOI:** 10.1186/s13287-025-04888-0

**Published:** 2026-01-07

**Authors:** Haiyuan Chen, Yazhu Zhong, Hao Zhang, Wei Yu

**Affiliations:** Key Laboratory of Respiratory Disease, People’s Hospital of Yangjiang, Yangjiang, Guangdong China

**Keywords:** Tissue renewal, Cell proliferation, Trachea, Stem cells

## Abstract

To elucidate spatiotemporal dynamics of tissue renewal, we developed evProTracer, an enhanced dual-recombinase lineage tracing system for cumulative in vivo labeling of proliferating cells. Robust longitudinal tracing using evProTracer in murine tracheal epithelium revealed near-complete homeostatic turnover (91.6 ± 1.29% epithelial replacement over 25 weeks), while basal cell-specific ​Trp63-evProTracer​ uncovered a dorsally biased proliferation pattern​, contributing 33.88 ± 1.44% of total epithelial renewal over 6 months, with early differentiation bias toward club cells. These data demonstrate that ventral epithelial renewal is primarily mediated by non-basal facultative progenitors, revealing their constitutive activation during homeostasis. This study uncovers spatially stratified renewal hierarchies: dorsal basal stem cell reservoirs versus ventral facultative non-basal progenitors. evProTracer provides a versatile platform for investigating tissue plasticity hierarchies in regenerative organs.

**Supplementary Information:**

The online version contains supplementary material available at 10.1186/s13287-025-04888-0.

## Introduction

The concept of tissue renewal was first proposed as early as 1956, when pioneering studies observed that multiple adult tissues exhibited remarkably high mitotic activity yet maintained stable tissue mass without progressive expansion [1]. Mammalian tissues exhibit distinct renewal patterns: rapidly renewing tissues (e.g., gut, skin) rely on stem cell-driven replacement (days-weeks) regulated by Wnt signaling [2]; slowly renewing tissues (e.g., liver, lung) maintain minimal baseline turnover (months) but activate robust regeneration after injury [3–5]; and static tissues (e.g., heart, brain) show negligible renewal (< 1%/year), relying on fibrotic repair [6–8]. Understanding the principles of tissue renewal holds transformative potential for regenerative medicine, as identifying and monitoring the cellular sources that fuel organ homeostasis and regeneration is fundamental to deciphering tissue plasticity.

The pseudostratified epithelium lining the human and mouse trachea is primarily composed of three major cell types: basal cells, ciliated cells, secretory cells (club cells) [9–11], and several rare cell populations, including ionocytes, neuroendocrine cells, tuft cells and hillock cells [12–15]. Among these, tracheal basal cells have been demonstrated to function as stem cells capable of both self-renewal and repopulating other cell lineages during homeostasis and regeneration [9]. Moreover, secretory cells in the trachea exhibit remarkable plasticity by differentiating into ciliated cells but limited self-renewal in homeostasis [10]. Instead, they undergo rapid proliferation during tissue repair and retain the capacity to dedifferentiate and replenish the basal cell compartment following basal cell deletion [16]. These observations suggest the existence of two distinct stem cell populations in the trachea. To determine whether these populations act as long-term, self-renewing stem cell reservoirs under steady-state conditions, more precise experimental systems are required for thorough investigation.

## Methods

All animal procedures were conducted under the approval of the Institutional Animal Care and Use Committee (IACUC) of Yangjiang People’s Hospital and the Yunnan University Animal Center. *Ki67-rox-stop-rox-iCre* mice were constructed from GemPharmatech (Nanjing, China). *Trp63-DreER* (NM‐KI‐190029), *R26-CAG-DreER* (NM‐KI‐200040), *R26-CAG‐lGFP* reporter (NM‐KI‐200045), *R26‐CAG-rGFP reporter* (NM‐KI‐233483) mice were purchased from the Shanghai Model Organism Center. Tamoxifen (Sigma-Aldrich, T5648-5G) was dissolved in corn oil to a final concentration of 10 mg/mL and administered to adult mice via intragastric gavage at a dose of 0.1 mg/g body weight, with two doses delivered at 48-hour intervals. All experimental procedures were conducted using established methods as previously reported [17]. Primary antibodies used in this study were as follows: goat anti-GFP (Abcam, ab6662), rabbit anti-GFP (Invitrogen, A21311), rabbit anti-GSS (Proteintech, 11037-2-AP), goat anti-E-Cadherin (R&D, AF748), rabbit anti-Krt13 (1:500, Abcam, AB92551), rabbit anti-P63 (CST, #13109), rabbit anti-CC10 (Abcam, ab213203), rabbit anti-Acetyl-Tubulin (CST, #5335). Secondary antibodies used were Alexa Fluor 488, 555, or 647-conjugated donkey anti-goat and donkey anti-rabbit with a dilution of 1:1000 (Invitrogen, A32814, A32790, A32794, A32816, A32849).

## Results

### Construction of EvProTracer for characterizing Spatiotemporal proliferation dynamics in mouse liver and trachea

**Fig. 1:**
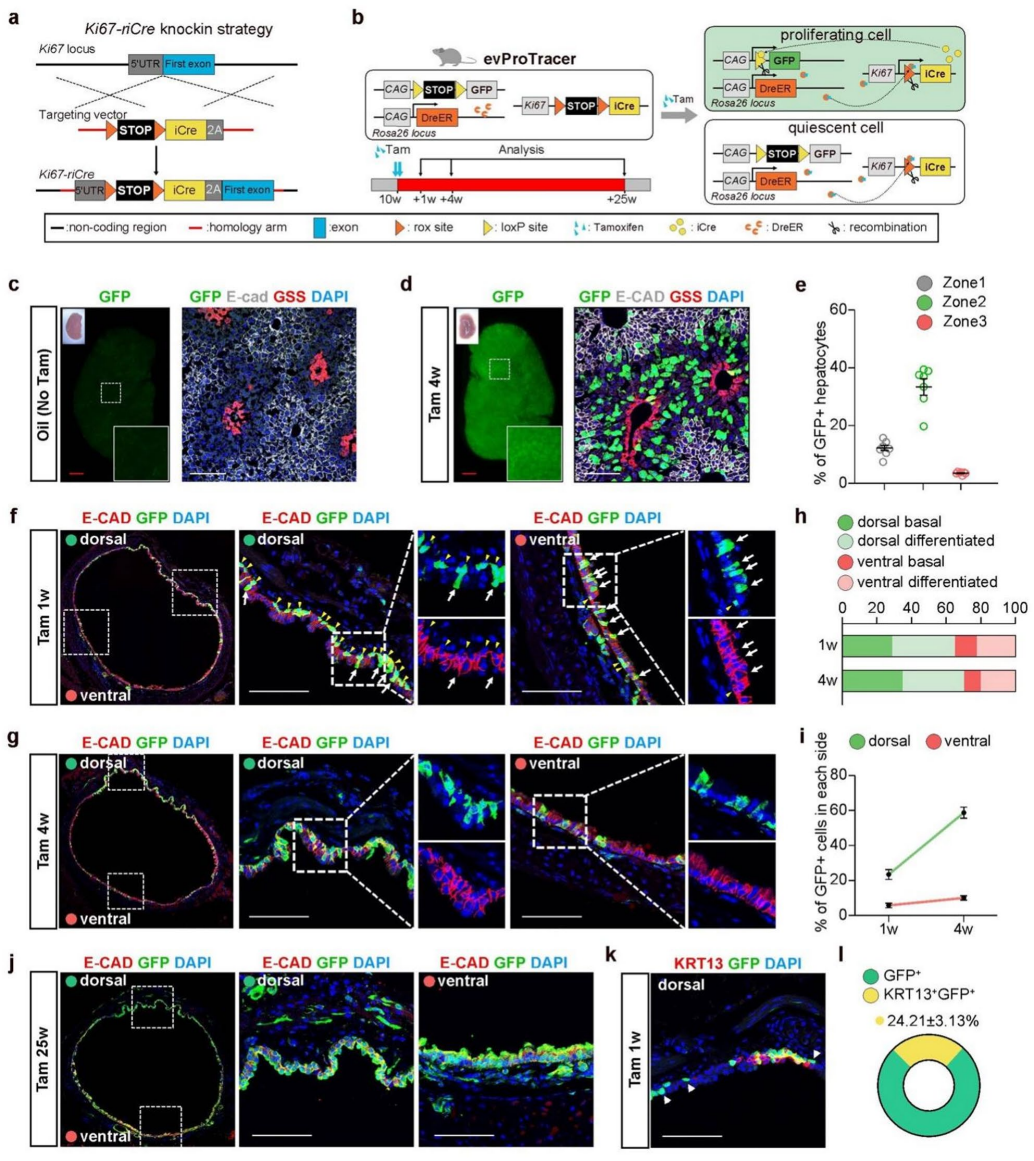
Characterization and application of evProTracer system for studying proliferative dynamics in liver and tracheal homeostasis.​​ **a** CRISPR-Cas9-mediated knock-in strategy for generating *Ki67-riCre* allele via homologous recombination. **b**​ Schematic of the evProTracer system for genetic recording of proliferating cells, including experimental timeline. **c**,** d** Whole-mount fluorescence and bright-field liver images at baseline (no Tam, **c**), and 4 weeks post-Tam injection (**d**); immunofluorescence staining of liver sections for GFP (evProTracer-labeled cells), glutamine synthetase (GS, pericentral), E-cadherin (E-CAD, periportal). **e​​** Quantitative distribution of GFP + hepatocytes across lobular zones: Zone 1 (portal, E-cadherin+), Zone 2 (midzonal, E-cadherin-GS-), Zone 3 (pericentral, GS+). **f**,** g** Tile-scan confocal images of tracheal epithelial sections from evProTracer mice stained for GFP and E-cadherin, visualized 1 week (**f**) and 4 weeks (**g**) post-Tam induction; high-magnification insets highlight the morphology of GFP⁺ cells. **h** Stacked bar charts illustrate the temporal and spatial dynamics of GFP + basal/differentiated epithelial cells in the dorsal/ventral at 1 week and 4 weeks post-Tam induction. **i** Quantification of GFP⁺ cell distribution across the dorsal/ventral sides of the tracheal epithelium at 1 week and 4 weeks post-Tam induction. **j** Tile-scan confocal image of tracheal sections co-stained for GFP and E-cadherin 25 weeks post-Tam, with dorsal/ventral magnified views. **k** Dual immunofluorescence staining for GFP/KRT13 in tracheal Sect. (1 week post-Tam). **l** Quantification of K13⁺GFP⁺ and GFP⁺ epithelial cell percentages (1 week post-Tam). White arrows: differentiated epithelial cells; yellow arrowheads: basal cells; white arrowheads: GFP + Krt13- cells. Scale bars: 1 mm (red, whole-mount); 100 μm (white, sections). All sections counterstained with DAPI (blue). Data presented as mean ± SEM; *n* = 3–6 in each group

To directly elucidate tissue turnover rates, we engineered a *Ki67-riCre* (*Ki67-rox-STOP-rox-iCre*) mouse line by inserting a *rox-STOP-rox-iCre* cassette into the endogenous *Ki67* locus, incorporating an improved Cre (iCre) with higher recombination efficiency than conventional Cre recombinase, as validated by prior methods [18] (Fig. 1a). Subsequently, we performed tripartite genetic crosses between the *Ki67-riCre* line, the tamoxifen-inducible Dre recombinase driver strain *R26-DreER* (*Rosa26-CAG-DreERT2*) and the Cre-responsive reporter line *R26-lGFP* (*Rosa26-CAG-loxP-STOP-loxP-GFP*). This breeding strategy generated compound transgenic mice with the genotype *Ki67-riCre; R26-DreER/lGFP*, which we designate as evProTracer (enhanced version Proliferation Tracer) in short (Fig. 1b). After tamoxifen (Tam) treatment, DreER undergoes nuclear translocation and mediates Dre-*rox* recombination to excise the rox-flanked STOP cassette in the *Ki67-riCre* allele, enabling constitutive iCre expression under the control of the endogenous *Ki67* promoter in DreER-expressing cells (Fig. 1b). Upon cellular proliferation, Cre-loxP recombination removes the loxP-flanked STOP in *R26-lGFP*, driving permanent GFP labeling. Without tamoxifen, evProTracer mice showed negligible liver GFP signals, confirming tight system control in the uninduced state (Fig. 1c).

Previous studies have documented a highly regionalized pattern of hepatocyte proliferation in the adult mouse liver midzone during homeostasis [5, 19]. To investigate whether evProTracer mice exhibit a similar proliferation pattern in the liver, we collected liver tissues at 4 weeks after Tam induction. A distinct GFP expression pattern was observed at the 4-week time point (Fig. 1d). To further validate these findings, we performed immunostaining using E-Cadherin as a marker for portal zones (Zone 1), Glutathione synthetase (GSS) as a marker for central zones (Zone 3), and GFP (proliferative cell marker) antibodies to identify proliferative hepatocytes across distinct lobular compartments. Quantitative analysis revealed a significantly elevated prevalence of ​GFP^+^ hepatocytes​ in ​Zone 2​ emerging as early as ​4 weeks post-Tam induction​ (Fig. 1e).

Recent studies show airway basal cells exhibit dorsal-ventral heterogeneity in injury response: dorsal basal cells display enhanced repair capacity and greater post-injury expansion, whereas there are no significant dorsoventral differences in Ki67⁺ cell ratios during homeostasis. In vitro analyses further reveal dorsal tracheal basal cells have higher primary culture colony-forming efficiency [20, 21]. Here we utilize evProTracer for in vivo cumulative lineage tracing of tracheal epithelium proliferation, establishing a spatiotemporal profile of cell division events during airway homeostasis. Following longitudinal lineage tracing, lung samples were harvested at 1-, 4-, and 25-week after tamoxifen induction. To ensure section comparability, we selected sections from cartilage rings 7 to 10 – a region of the thoracic trachea reported to contain the intercartilaginous niche – for further analysis [22, 23]. To spatially resolve dorsoventral heterogeneity, tracheal samples were transversely sectioned and subjected to confocal tile-scan imaging. Our data reveal a modest dorsal-biased distribution of GFP signals at 1 week post-induction, with 65.14 ± 2.46% of GFP-positive cells localized dorsally (Fig. 1f and h). This dorsal predominance became more pronounced by 4 weeks post-induction, with 70.32 ± 1.8% of GFP-labeled cells concentrated on the dorsal side (Fig. 1g and h). Additionally, at 4 weeks post-induction, a stacked bar chart revealed an approximately equal distribution of GFP⁺ cells between basal and differentiated phenotypes in the dorsal compartment. By contrast, in the ventral side, the majority of GFP^+^ cells exhibited a differentiated epithelial phenotype, suggesting that proliferating cells in the ventral region are primarily differentiated epithelial cells (Fig. 1h). We further quantified epithelial renewal rates in both compartments and observed that GFP⁺ cells in the dorsal region exhibited a marked increase, comprising 58.8 ± 3.15% of labeled cells at 4 weeks post-induction. In contrast, only 10.07 ± 1.24% of epithelial cells in the ventral region displayed GFP labeling at this time point (Fig. 1i). This discrepancy emphasizes the dorsal proliferation bias. Furthermore, long-term cumulative tracing revealed that 91.6 ± 1.29% of tracheal epithelial cells acquired lineage labels over 25 weeks of monitoring (Fig. 1j). This finding demonstrates that the murine tracheal epithelium undergoes near-total replenishment within a 6-month cycle, defining a basal turnover rate under homeostatic conditions. Hillock cells have recently been reported to be uniquely proliferative cells that drive high-turnover squamous cell populations during homeostasis [24]. Dual KRT13 (hillock marker) and GFP immunostaining of evProTracer tracheal sections at 1 week post-induction revealed 24.21 ± 3.13% of GFP⁺ cells co-expressed KRT13 (Fig. 1k and l), confirming hillock cells contribute to tracheal turnover, and non-hillock proliferative populations also play an active role in maintaining tracheal homeostasis.

### Trp63-evProtracer unmasks dorsal-enriched basal cell dynamics in tracheal homeostasis​

**Fig. 2:**
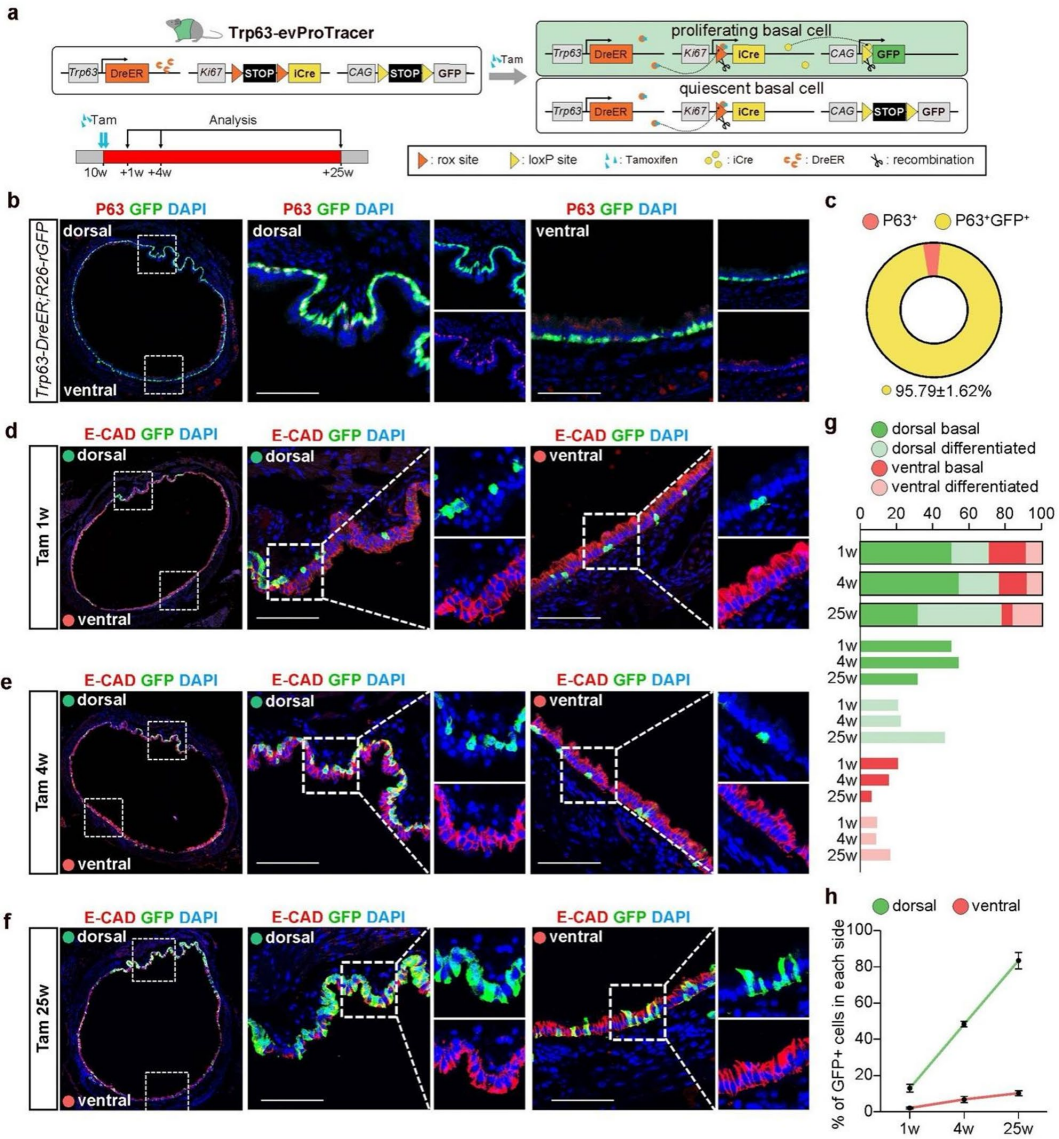
Longitudinal recording of basal cell proliferation dynamics in airway epithelium via Trp63-evProTracer. **a** Schematic of the Trp63-evProTracer system for basal cell-specific proliferation recording. **b** Tile-scan confocal images and magnification views of adult *Trp63-DreER; R26-rsrGFP* tracheal Sect. (4 days post-Tam induction), co-stained for GFP and P63 (basal marker). **c** Pie chart showing GFP⁺ basal cell proportion in *Trp63-DreER; R26-rsrGFP* trachea (4 days post-Tam). **d-f** Tile-scanned confocal microscopy of Trp63-ProTracer tracheal sections co-stained for GFP (green) and E-cadherin (red) at 1 week (**d**), 4 weeks (**e**) and 25 weeks (**f**) post-Tam induction ​with​ magnified views of dorsal and ventral regions. High-magnification insets highlight the morphology of GFP⁺ cells. **g** Stacked bar charts illustrating temporal and spatial dynamics of GFP + basal/differentiated epithelial cells (dorsal/ventral, 1/4/25 weeks post-Tam induction), with layered bars for category comparisons. **h** Quantification of the percentage of GFP⁺ cells in dorsal and ventral sides of the Trp63-ProTracer tracheal epithelium (1/4/25 weeks post-Tam induction). Scale bar: 100 μm. Data shown are mean ± SEM; *n* = 3–5 for each group

To further validate the proportion of proliferating tracheal epithelial cells originating from basal cells under steady-state conditions, we generated Trp63-evProTracer (*Trp63-DreER; Ki67-riCre; R26-lGFP*) through crossing *Trp63-DreER* (basal cell-specific DreER mice) with *Ki67-riCre; R26-lGFP* mice (Fig. 2a). Using *Trp63-DreER; R26-rGFP* reporter mice, we confirmed even tracheal distribution of P63⁺ basal cells via lineage labeling (short-term Tam induction) and immunostaining, consistent with prior work (Fig. 2b) [20]. Quantitative analysis confirmed high labeling specificity and efficiency in *Trp63-DreER* mice (95.79 ± 1.62% GFP^+^ basal cells) (Fig. 2c). Subsequent longitudinal lineage tracing employed this system, with systematic collection of lung specimens at 1-, 4-, and 25-week intervals post-tamoxifen administration in line with prior methodologies (Fig. 2a). Confocal tile-scan imaging revealed GFP-labeled basal cells exhibited sustained dorsal predominance throughout the 25-week tracing period (Fig. 2d and f). Quantitative analysis using stacked bar charts confirmed this pronounced dorsal bias in GFP⁺ cell distribution (Fig. 2g). Besides, during early phase (1–4 weeks), GFP labeled cells predominantly maintained basal morphology, whereas long-term cumulative tracking (25 weeks post-induction) demonstrated their progressive differentiation into epithelial cells in both compartments (Fig. 2d and g). We further quantified the percentage of GFP⁺ cells in each side and found that dorsal epithelial cells exhibited a marked renewal dynamic. Specifically, GFP⁺ cells increased dramatically from 13.03 ± 2.06% at 1 week to 83.46 ± 4.47% by 25 weeks post-induction. In contrast, GFP labeling in the ventral compartment remained low, reaching only 10.3 ± 1.24% at 25 weeks (Fig. 2h). Critically, basal lineages contributed merely 33.88 ± 1.44% of total epithelial renewal over 6 months, indicating substantial involvement of alternative non-basal progenitor populations in tracheal homeostasis.

To track the differentiation trajectory of GFP-labeled cells, we further conducted immunostaining for GFP, CC10 (club cell marker), and acetylated tubulin (A-tubulin, ciliated cell marker) in tracheal sections at 1-week, 4-week, and 25-week post-tamoxifen time points. At 1 week, GFP signals were predominantly restricted to basal cells, with no detectable differentiation toward airway secretory or ciliated lineages (Fig. S1a and c). By 4 weeks post-tam, a distinct subset of GFP⁺ cells had differentiated into CC10⁺ club cells; however, GFP⁺ ciliated cells remained nearly undetectable, indicating a preferential early differentiation bias toward the club cell lineage (Fig. S1a-b). Notably, at the 25-week long-term tracing time point, GFP⁺ ciliated cells became identifiable, yet they only constituted less than 10% of the total ciliated cell population, reflecting a slow and limited differentiation potential of labeled cells toward the ciliated lineage (Fig. S1a-d). Additionally, we assessed hillock cell turnover in long-term lineage tracing sections by immunostaining for KRT13. Quantitative analysis revealed that 82.2 ± 5.19% of total KRT13⁺ hillock cells underwent renewal within the 25-week tracing window (Fig. S1e-f). Collectively, our data demonstrate that dorsal Trp63⁺ basal stem cells possess markedly higher self-renewal activity and stronger lineage differentiation potential compared with their ventral counterparts.

## Discussion

In summary, this study introduces ​evProTracer, an enhanced proliferation tracing system enabling cumulative spatiotemporal monitoring of in vivo proliferative dynamics. Our findings redefine tracheal homeostasis through a spatial hierarchy model: dorsal regions rely on basal stem cell self-renewal, while ventral compartments utilize facultative non-basal progenitors. The ​evProTracer platform​ overcomes fundamental limitations of pulse-chase methods by enabling cumulative proliferation recording—critical for quantifying slow-cycling populations. Its application revealed near-complete tracheal epithelial turnover (91.6 ± 1.3% over 25 weeks) in the adult mouse trachea, which aligns with prior lineage tracing studies using *FoxJ1-CreER*, documenting a progressive dilution of ciliated epithelial cells over 7-month period [11]. Trp63-evProTracer provides the first in vivo evidence of dorsal-enriched basal cell dynamics, with these cells contributing 33.88% to total epithelial renewal. Cell fate analysis clarified that dorsal basal cells exhibit an early differentiation bias toward CC10⁺ club cells (with GFP⁺ ciliated cells undetectable at 1–4 weeks), while 82.2 ± 5.19% of K13⁺ hillock cells renewed within 25 weeks (validating their role in high-turnover tracheal subsets), collectively confirming dorsal Trp63⁺ basal cells have superior self-renewal and differentiation potential compared to ventral counterparts. Notably, this finding indicates that ventral facultative progenitors—likely of secretory/club cell origin—also exhibit constitutive activation under steady-state conditions. This sustained activity in ventral progenitors may be driven by shear stress and epithelial loss, stemming from the continuous movement of the underlying posterior membrane induced by dorsal breathing motions. Beyond airway biology, evProTracer provides a methodological framework to investigate ​niche-specific progenitor hierarchies​ in other regenerative organs, with implications for understanding region-selective disease vulnerabilities.

## Supplementary Information

Below is the link to the electronic supplementary material.

Supplementary Material 1

## Data Availability

No datasets were generated or analysed during the current study.
