# Supplementary figures and images for: Enhanced proliferation tracer reveals Dorsal-Ventral asymmetry in tracheal epithelial Renewal​
Source: Stem Cell Res Ther. 2026 Jan 7;17:69. doi: 10.1186/s13287-025-04888-0 (PMC12870089; doi:10.1186/s13287-025-04888-0)

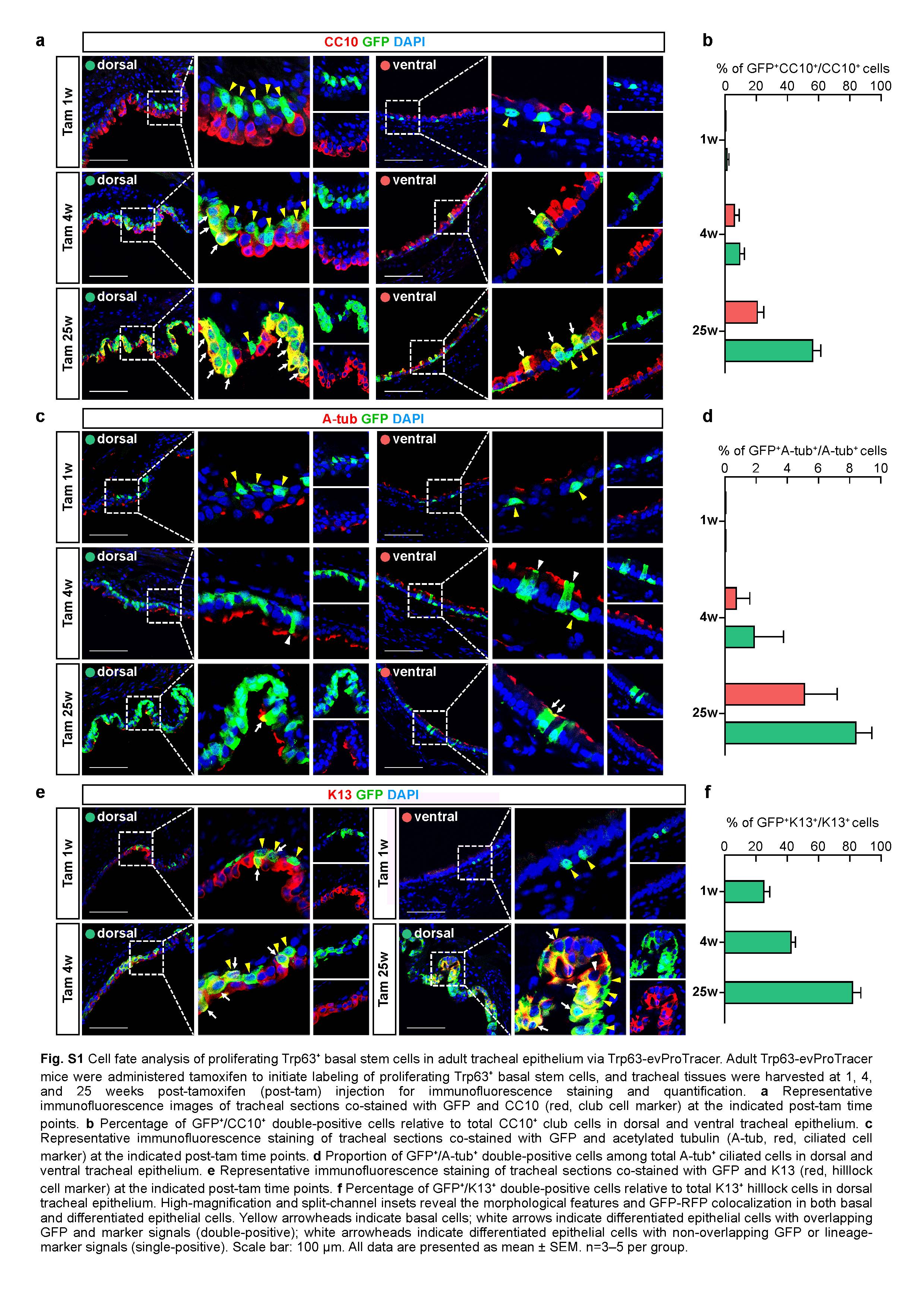

Supplement: Supplementary file 1 — Supplementary Material 1 [file 13287_2025_4888_MOESM1_ESM.jpg]
